# Pressure Drop Dynamics during Filtration of Mixture Aerosol Containing Water, Oil, and Soot Particles on Nonwoven Filters

**DOI:** 10.3390/polym15071787

**Published:** 2023-04-04

**Authors:** Mateusz Kamiński, Jakub M. Gac, Piotr Sobiech, Paweł Kozikowski, Tomasz Jankowski

**Affiliations:** 1 Faculty of Chemical and Process Engineering, Warsaw University of Technology, ul. Waryńskiego 1, 00-645 Warsaw, Poland; 2 Central Institute for Labour Protection-National Research Institute, ul. Czerniakowska 16, 00-701 Warsaw, Poland

**Keywords:** fibrous filter, filtration efficiency, mixture aerosol, pressure drop, wettability difference

## Abstract

The pressure drop dynamics during the filtration of three-component mixture aerosols are investigated and compared with two and single-component aerosols. The main area of interest is the effect of the addition of a small quantity of liquid (oil) and solid (soot) particles during the filtration of aerosol containing water mist. In addition, calculations of the change in filter mass during oil aerosol filtration have been carried out and compared with the experimental results. The new, improved filtration efficiency model takes into account a better coefficient fitting in the filtration mechanism equations. The limitations in the change in fibre diameter and packing density resulting from the filter loading have been implemented in the model. Additionally, the calculation model employs the fibre size distribution representation via multiple average fibre diameters. The changes in fibre diameter are dependent on each fibre’s calculated filtration efficiency. The improved filtration model has been utilised to predict the mass change of the filters during the filtration of pure and mixture aerosols. The pressure drop calculation model based on changes in filter mass has been formulated. The model is then utilised to calculate pressure drop changes resulting from the filtration of the oil aerosol and water and oil mixture aerosol.

## 1. Introduction

Fibrous filters are widely utilised in many industrial applications [1,2,3], environmental protection [4,5], and healthcare [6,7,8]. They are used to remove both solid and liquid particles from gas streams. A logical development of the described topic is the issue of the three-component aerosol filtration, i.e., consisting of solid particles and drops of two types of liquid that do not mix with each other, e.g., oil and water. This problem may be of great practical importance, especially in the case of engine exhaust filtration, where apart from solid soot aggregates, the water droplets resulting from the condensation from combustion and drops of unburned liquid fuel may appear [9]. Additionally, in the oil industry, a coexistence of oily liquids (crude oil), water, and solid particles from the parent rock may appear [10]. This work is the first attempt to describe the filtration process defined in this way.

The reason for the high demand for fibrous filters is their very high efficiency, which often exceeds 90%, and the relatively low cost of production. The extensive knowledge of the phenomena occurring inside these filters is also important, which allows the design of an appropriate nonwoven structure defined primarily by the porosity of the filter, the size distribution of fibres, and the material from which the fibres are made. Importantly, it does not have to be a homogeneous structure: often, the use of multi-layer or gradient filters gives better results or leads to easier modifications/alterations for specific usage [11,12]. As the production technologies allow for the production of such designed structures, it opens the way for further improvement of the separation properties of fibrous filters.

The first works on filtration on nonwoven filters, the efficiency of these filters, pressure drops, and the evolution of both of these values during filter operation dealt with the filtration of solid particles [3,13]. As part of these studies, the classical filtration theory (CFT) has been formulated, allowing to determine the filtration efficiency of particles of particular sizes depending on the average diameter of the filter fibres and the linear velocity of the gas [14,15]. More advanced studies on filtration efficiency have utilised more indirect simulations. There is a large number of studies describing the gas flow field in the filter by means of computational fluid dynamics (CFD) and the particle–fibre interactions by means of the discrete element method (DEM) [16,17,18]. Other methods for filtration efficiency simulations were based on the lattice Boltzmann method [19,20].

A description of the formation of filtration aggregates and their influence on changes in filtration efficiency and flow resistance through the filter was also formulated, both in terms of experimental studies and numerical simulations [21,22]. The formed aggregates increase the surface area of the fibres. Such an effect leads to an increase in filtration efficiency. At the same time, by reducing the porosity of the filter, they caused an almost monotonic increase in the flow resistance through the filter [23,24]. In subsequent studies, much attention was also paid to the phenomenon of re-entrainment, i.e., the secondary entrainment of deposited aerosol particles (individual particles or fragments of filter deposits) into the flowing gas stream [25,26]. This phenomenon resulted in an effective decrease in the filtration efficiency (secondary entrained particles appeared in the outlet stream) or a modification of the mass distribution of the retained dust inside the filter.

The next stage of filtration research on nonwoven filters was related to droplet filtration. It was quickly noticed that in the case of droplets with sizes below 10 µm, the initial filtration efficiency is described by the same relationships as the filtration efficiency for solid particles obtained from the classical theory of filtration [27]. However, the dynamics of filtration efficiency, as well as the dynamics of pressure drop, are completely different for both solid and liquid particles. This is due to a completely different nature of the liquid retained in the filter structure. While in the case of the filtration of solid particles, fractal-like, highly developed, but equally highly porous filtration aggregates were formed; the droplets formed relatively homogeneous layers surrounding the filter fibres. As more and more liquid accumulates, these layers join together to form liquid bridges, which constitute a very high resistance to the flowing gas [28]. This resulted in a characteristic shape of the dynamics of the pressure drop—after the initial, relatively smooth increase, there was a rapid, several-fold jump in the pressure drop [28,29]. Then, a state was developed close to dynamic equilibrium—the amount of liquid retained by the filter was balanced by the amount of liquid entrained from the filter, and the pressure drop did not experience any major changes.

Another development on the subject of filtration on nonwoven filters was the issue of the filtration of mixture aerosols, i.e., aerosols containing both solid particles and liquid drops. In one of the first studies [30], the dynamics of pressure drop during the filtration of such aerosols has been investigated. One of the main conclusions was the sharp jump in the flow resistance that is highly characteristic of mist filtration. Ref. [31] investigated the effect of oil deposited on filter fibres on the efficiency of the removal of solid particles from a gas stream. In contrast, in the work by [32], the influence of soot particles on the filtration efficiency of liquid aerosols was investigated. There are also studies on the alternating (consecutive) filtration of solid and liquid particles [33,34]. These works theoretically and experimentally observed as well as numerically described the collapse of the filtration deposits under the influence of the deposited liquid, which resulted in a decrease in filtration efficiency and flow resistance during such a process compared to the filtration of only solid particles. A simultaneous filtration of solid and liquid particles was investigated in our last work. By examining the simultaneous filtration of soot particles and water droplets [35], as well as soot particles and oil droplets [36], we observed similar effects as in the case of alternate filtration: the collapse of the filter deposits leading to a decrease in efficiency and the evolution of the pressure drop similar to the filtration of liquid aerosols.

However, it should be emphasised that in the vast majority of the mentioned theoretical and numerical works, the authors focus mainly on determining the filtration efficiency and its evolution, ignoring the size of the pressure drop and its changes. The methods of determining the initial pressure drop, i.e., for unloaded filters, were presented in Davies’ fundamental work [37] and the approximate formulas presented there, together with the concept of equivalent “Davies diameter”, are utilised even today. Wang et al. [38] used stochastic modelling to determine the pressure drop values for filters of different porosity and thickness, obtaining similar results as from the approximate Davies equations. In [39], the pressure drop of nonwoven filters has been obtained by the means of the Hagen–Poiseuille equation for gas flow through porous materials. Changes in pressure drop due to filter deposits build-up has been addressed by Thomas et al. [23] using an approximate method that treats filter deposits as additional fibres that are present in the filter structure. When analysing the evolution of pressure drop during liquid aerosol filtration, one should note the works by Frising et al. [40] and Payet et al. [41] along with utilising a semi-empirical model work by Gac. When considering the filtration of mixed solid–liquid aerosols, to our knowledge, there are currently no papers numerically or analytically describing the changes in pressure drop during such a process.

Our current work is the next step in the topic of mixture aerosol filtration. We move from binary systems to ternary systems containing solid particles and liquid particles of two different liquids. The results of experimental research will be supported by an analytical description, which is a certain extension of Davies’ description for the case of filtration of binary and ternary aerosol systems.

## 2. Materials and Methods

### 2.1. Experimental Setup

**Figure 1:**
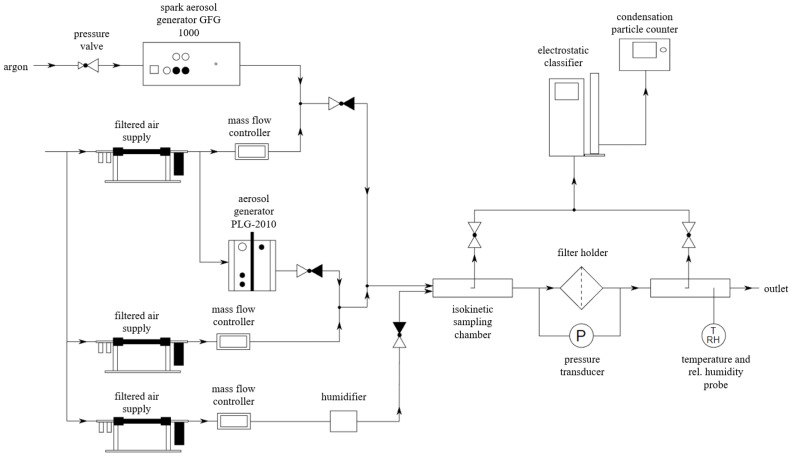
Experimental setup.

The setup of three independently operating generators was utilised (Figure 1). The GFG 1000 (Palas GmbH, Karlsruhe, Germany) spark discharge aerosol generator was utilised to generate solid particles aerosol. The graphite electrode with a density of 2090 kg/m^3^ was utilised with the generator. The obtained nanostructured graphite particles will be referred to as soot due to their similarities in morphology. The PLG-2010 (Palas, Germany) Laskin nozzle aerosol generator was utilised to generate oil aerosol. The DEHS (di-ethylhexyl-sebacate) oil of a density of 914 kg/m^3^ was utilised for that purpose. The membrane humidifier, placed in a metal tank with an air inlet and connector to a horizontal tank, was utilised to generate water mist. Additionally, the dry air (5% humidity) of adequate flow was utilised to ensure steady aerosol flow. Compressed air used for test aerosol generation was filtered using Filtered Air Supply 3074B (TSI Inc., Shoreview, MN, USA). The average flow velocity of the aerosol upstream from the filter was 0.2 m/s and was the same for all the experiments and independent of the aerosol composition. The temperature of the aerosol stream was around 295 K. The filter samples utilised in experiments were disc-shaped, with their primary diameter being 100 mm and the part exposed to the aerosol flow being 80 mm. The contact area between the aerosol stream and the sample was 50.3 cm^2^. Graphite nanoparticles and DEHS particles concentration, distribution, and filter efficiency were determined using a scanning mobility particle sizer spectrometer (SMPS) comprising ultrafine condensation particle counter (UCPC 3776) and electrostatic classifier 3082 (TSI Inc., USA). Spraytec laser diffraction system (Malvern Instruments Ltd., Malvern, UK) was used to obtain the number size distribution of water droplets. Pressure drop across filters was measured using a P26 differential pressure transducer (Halstrup Walcher GmbH, Kirchzarten, Germany).

### 2.2. Filters

**Figure 2:**
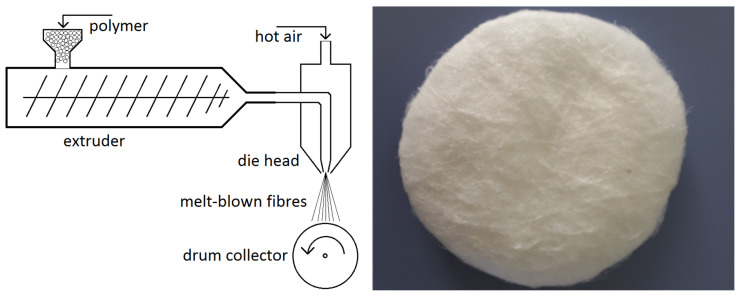
Melt-blown method overview (**left**) and obtained filter sample (**right**).

The material utilised for layer production was polypropylene, and layers were obtained by melt-blown fabrication method (Figure 2). This method involves extruding molten polymer through the head holes surrounded by hot air, the flow of which helps to achieve the desired fibre size. Contact with cold air leads to the cooling of the polymer streams and the formation of fibres, which are then collected by a collector to form a layer of a given thickness (Figure 2). The average fibre diameter was calculated based on SEM (scanning electron microscope-HITACHI SU8010) images of filter samples. There were 120 fibres analysed for each filter, and around 20 images in different parts of multiple samples were taken. The thickness of each fibre was measured in three different parts of the fibre, and the obtained average value of the number of pixels for each fibre was then converted to the actual size based on the scale. The layer thickness was measured with a Tilmet-79 thickness gauge (high contact area) with an accuracy of 0.01 mm, and 20 measurements for each filter. The packing density was calculated based on sample weight and dimensions.

### 2.3. Aerosols

**Figure 3:**
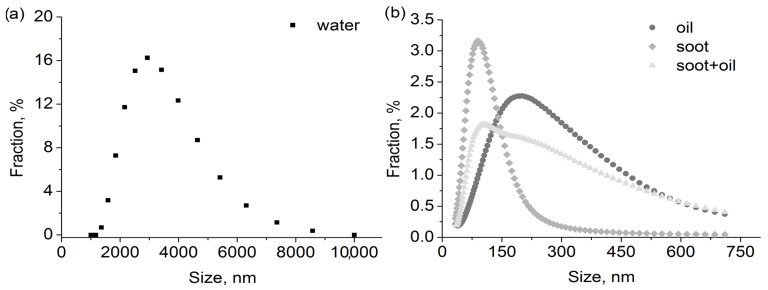
Particle size distribution for (**a**) water and (**b**) oil and soot aerosols.

Table 1 lists the properties of pure aerosols. Additionally, mixtures of these aerosols were utilised in experiments. In the case of mixture aerosols, water would be responsible for the vast majority of the mass captured by the filter due to larger particle sizes and mass flow. As shown in Figure 3, the entirety of the oil aerosol particle size distribution could not be measured due to equipment limitations. Oil and water aerosols were chosen for their differences in wettability towards filter material. In the case of a mixture of soot + oil aerosol, the total concentration is lower than the sum of components due to particle interactions before the aerosol reaches the filter. The average particle size is a weighted arithmetic mean (by share).

### 2.4. Efficiency and Mass Changes Calculations

The filtration mechanism of diffusion, interception, and impaction were included in the calculation model. The electrostatic mechanism was omitted due to the filtration material not being charged with static charges (samples were neither intentionally charged nor discharged; however, the existence of some charges related to the manufacturing process may still be possible). The filtration efficiency calculations and its changes over time were based on the following empirical equations which were discussed in detail in our previous papers [36,42]:

(1) $$\eta_{dif}=A\cdot {Pe}^{-B}$$

(2) $$\eta_{int}=C\left(\frac{1-\alpha }{Ku}\right)\left(\frac{R^{2}}{1+R}\right)$$

(3) $$\eta_{imp}=D\cdot {Stk}^{E}$$

where η_dif_—diffusion mechanism efficiency (-), Pe—Peclet number (-), η_int_—interception mechanism efficiency (-), α—packing density (-), Ku—hydrodynamic factor of Kuwabara flow (-), R—interception parameter (-), η_imp_—impaction mechanism efficiency (-), and Stk—Stokes number (-).

The total filter efficiency was calculated based on the fractional efficiency towards aerosol particles and particle size distributions of the tested aerosols. For that purpose, the following equations for filter efficiency and single fibre efficiency were utilised:

(4) $$\eta =1-(1-\eta_{dif})(1-\eta_{int})(1-\eta_{imp})$$

(5) $$E=1-exp\left(-\frac{4\cdot \eta \cdot \alpha \cdot Z}{\left(1-\alpha \right)\cdot \pi \cdot d_{f}}\right)$$

where η—calculated single fibre efficiency (-), E—filter efficiency (-), α—packing density (-), Z—filter thickness (m), and $d_{f}$—fibre diameter (m).

The values of the coefficients A, B, C, D, and E have been adjusted accordingly for each filter to increase the accuracy of the model. The same coefficients are applied to each pair of filters. The values were determined based on measured initial efficiency during the filtration of oil aerosol. 

The changes in efficiency over time were dependent on the volume of deposits captured by the fibres. For that purpose, for each filter, the fibre size distribution was prepared via scanning electron microscope images. The measured distribution was reduced to ten average values, each representing one-tenth of all fibre diameters. This allowed the simplification of the model without significantly affecting the accuracy [42]. The calculations of efficiency change over time were performed for each of the fibre diameters independently, and their contribution to the total filter efficiency was weighed based on their share. The details of this method were described in our previous work [42].

The number of the captured particles was converted into their volume and subsequently into a change in packing density, average flow velocity inside the filter, and fibre diameter. Some restrictions were implemented to account for the accumulation of captured particles in the dead zones of the filter, the re-entrainment of the droplets, as well as the presence of a dynamic equilibrium between liquid particles being captured by the fibre and the movement of the liquid on the surface of the fibre:

- The maximum allowed change in fibre diameter (25% increase);
- The portion (15%) of the volume effectively influencing change in packing density in each time step.

The calculations were performed with the time step of 1 s, and the total time of predicted changes using the model was 3840 s.

### 2.5. Pressure Drop Changes Calculations

Similar to changes in efficiency, pressure drop changes were calculated based on changes in average flow velocity, packing density, and fibre diameters. The following equation was utilised in pressure drop calculations:

(6) $$P=F\cdot \mu \cdot u\cdot Z\cdot \frac{\alpha^{G}}{d^{H}}$$

where P—pressure drop (Pa), µ—fluid viscosity (Pa·s), u—average flow velocity in a filter (m/s), Z—filter thickness (m), α—packing density (-), and d—fibre diameter (m).

For each tested filter, values of the coefficients F, G, and H were determined based on the filter properties before and after the filtration of oil aerosol. The same restrictions on changes as for efficiency and mass calculations have been applied for pressure drop changes calculations. The obtained equations were then utilised to calculate the changes during the filtration of the mixture aerosol.

In reality, the fluid viscosity depends on the composition and concentration of aerosol particles for sufficiently high concentrations. The average linear flow velocity of the fluid depends on the packing density and the average fibre diameter, which will vary dynamically during filtration. This change will occur non-uniformly throughout the thickness of the filter and is strongly related to the aerosol properties. As for now, Equation (6) treats the entire filter layer as homogeneous at each time step. It does not take into account the mass distribution in the interior of the filter and changes in this distribution with time.

## 3. Results

### 3.1. Filter Properties

**Figure 4:**
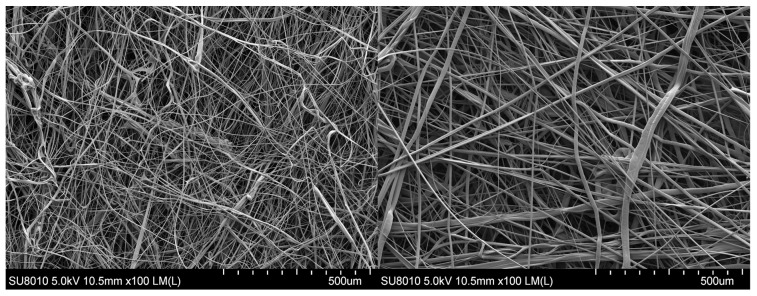
Structure of the (**left**) F1 filter and (**right**) F6 filter.

**Figure 5:**
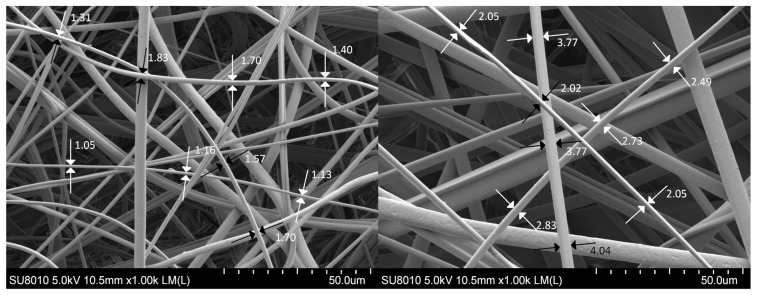
Example of the utilised measurement method for the (**left**) F1 filter and (**right**) F6 filter.

The nonwoven filters were analysed by SEM and the results are presented in Figure 4. The SEM analysis revealed that the filters possess a homogenous and uniform microstructure, with randomly arranged fibres. Additionally, the filters exhibited fibres of varying sizes. The determination of fibre size distribution was carried out using the measurement method depicted in Figure 5.

**Figure 6:**
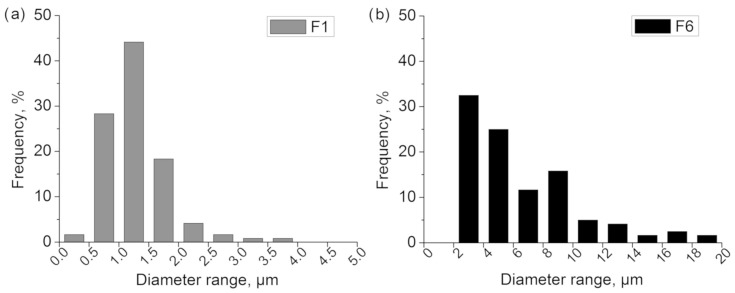
Measured fibre size distribution for (**a**) F1 filter and (**b**) F6 filter.

Table 2 shows the properties of filtration layers utilised in experiments. The designations of the F6 and F1 filters correspond to the average diameter of the fibres which the particular layer comprises. The layers marked as 2F6 and 2F1 consist of the same fibres as layers F6 and F1, but their thickness and mass are higher. The layer thickness of the F6 filter is clearly lower in comparison with any other tested filter. The 2F1 and F1 layers are characterised by a lower value of average fibre diameter and a higher value of pressure drop in comparison with 2F6 and F6 layers. The 2F6 and F1 filters have similar thickness. The average fibre diameter and its deviation are weighted arithmetic means (by share). Figure 6 shows the obtained fibre size distributions. The fibre size distribution for the F1 filter is more narrow than the one for the F6 filter.

### 3.2. Calculated Oil Droplets Distribution

As previously mentioned, droplets larger than ~750 nm could not be measured due to equipment limitations. However, they cannot be ignored in the calculation of the filter loading. In order to include their presence in the stream in the calculations, modelling was carried out to complete the distribution. The exponential function based on the sizes between 400 and 710 nm was utilised for that purpose. The authors decided not to utilise the distribution fitting method due to the asymmetry of the distribution and its overall shape resulting partially from particle interactions (Smoluchowski’s coagulation equation would be a more fitting and precise solution, yet is far more complicated to implement). Only the right side of the distribution was modelled. The smallest droplets were omitted due to their insignificant contribution to the change in filter characteristics. Figure 3 shows the results of the calculations. 

**Figure 7:**
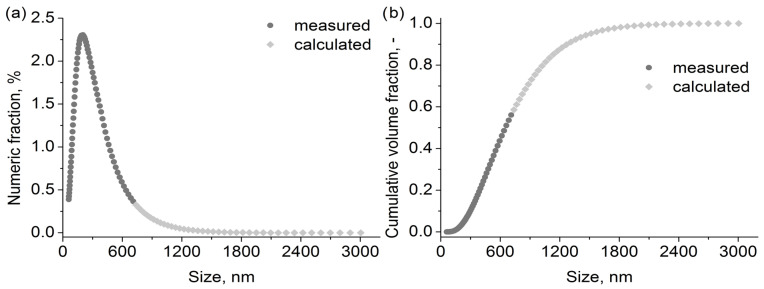
Calculated (**a**) numeric fraction and (**b**) cumulative volume fraction for oil aerosol.

Despite the small numeric fraction (Figure 7a), the additional droplets have a significant impact on the volume and mass of deposits retained on the filter due to their large size. The calculation results indicate that the droplets in the modelled range account for more than 40% (Figure 7b) of the total volume present in the aerosol stream during the oil aerosol filtration.

### 3.3. Calculated Efficiency and Mass Changes

**Figure 8:**
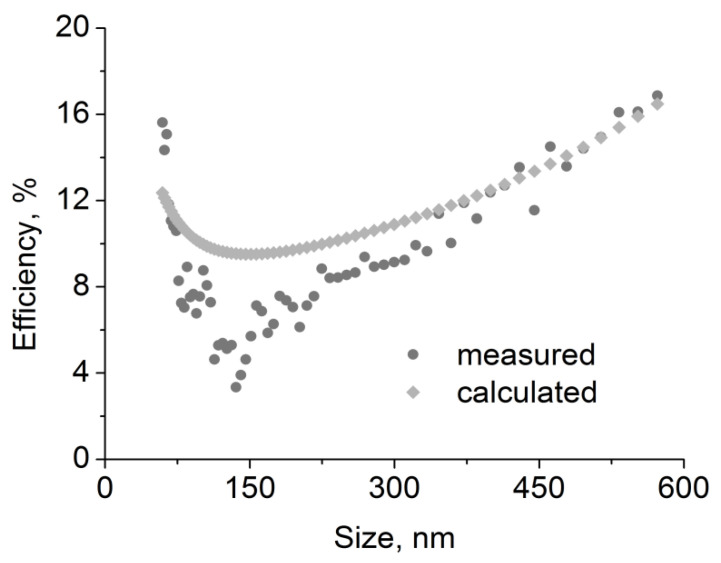
Measured and calculated fractional efficiencies for oil aerosol for 2F6 filter.

Figure 8 shows the reasonably accurate results of the coefficients fitting for the 2F6 filter. The main consideration was to make the suitable fitting for the larger droplets, and thus the most relevant to the changes taking place inside the filter. Sizes below MPPS (most penetrating particle size) are less relevant due to their limited contribution to the changes occurring inside the filter.

In the case of other filters, the differences between calculations and measurements are slightly greater than for the 2F6 filter (even though it is still of a similar order of magnitude, and there is a clear minimum of around 100–200 nm). However, this is not of great importance for further calculations because, as shown below, the dynamic of filter loading is significantly influenced by particles and droplets with diameters significantly exceeding the range presented in Figure 8. 

Table 3 shows the obtained values of the mechanism efficiency coefficients for the tested filters for the oil aerosol. The coefficients were utilised to calculate the change in filter mass during the 3840 s long filtration process. The results for all filters are shown in Table 4. 

The predicted mass change for the 2F6 filter is consistent with the measured value, and is considerably more accurate than the previous model, which did not take into account the presence of larger droplets. Similarly, in the case of the 2F1 and F1 filters, the consideration of additional droplets in the model and the new values of the coefficients clearly improved the agreement of the measured and calculated results with respect to the previously applied model. 

While the higher mass change compared to the previous model is not surprising (we take into account the wider range of droplets), the discrepancy between the measured and calculated values in the case of the F6 filter is significant. This can be explained by the lower layer thickness of this particular filter. Interactions between fibres and large droplets which are destructive for the latter are not included in the current model. This can lead to a lower chance of further interactions and a lower actual efficiency for this filter. A similar effect could occur in the case of interactions between droplet and wet fibre. The high overall efficiency of 2F1 and F1 filter results from capturing occurring in the upstream side part of the filter. This results in a non-uniform change of properties within the filter, and the differences will be more significant the higher the filter efficiency. The current model does not take this effect into account.

### 3.4. Measured and Calculated Pressure Drop Changes for Oil Aerosol

**Figure 9:**
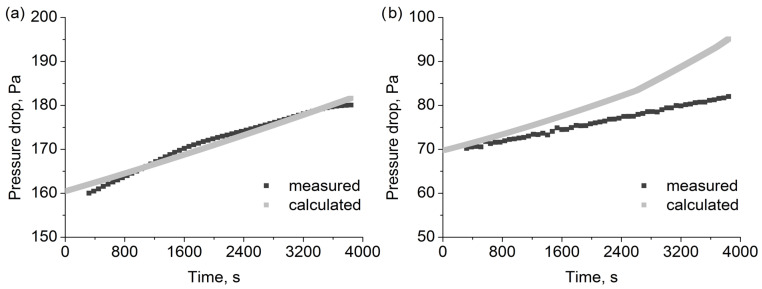
Pressure drop changes calculated and measured for (**a**) 2F6 and (**b**) F6 filters during oil aerosol filtration.

**Figure 10:**
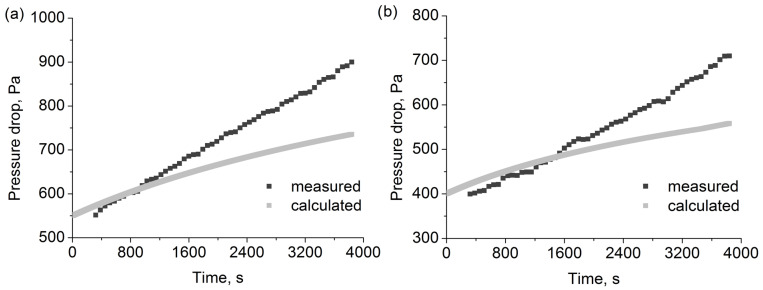
Pressure drop changes calculated and measured for (**a**) 2F1 and (**b**) F1 filters during oil aerosol filtration.

Table 5 shows the obtained values of the pressure drop coefficients for the tested filters for the oil aerosol. The coefficients were utilised to calculate the change in pressure drop during the 3840s long filtration process. The results for all filters are shown in Figure 9 and Figure 10. Utilising the determined values of the coefficients, we calculated the change in pressure drop over time. The first 320s were omitted to ensure stable readings after changes in flows resulting from the start-up of the aerosol generators. At the same time, the calculations start from the zero time point.

As the pressure drop calculation model is dependent on the filter efficiency model via changes in packing density, average flow velocity, and fibre diameter resulting from the capture of the particle droplets, the pressure drop change over time ought to be as accurate as mass change over time. The most accurate results were obtained for the 2F6 filter, for which the mass change prediction was also the most accurate. For the F6 filter, the calculated pressure drop change was higher than the measured one. Additionally, after the calculation time of the 2700 s, the change in fibre diameter reached the maximum allowed value (for the most efficient fibres), and the higher rate of change in pressure drop after that time point can be observed in the calculation results. For both the 2F1 and F1 filters, the calculated change in pressure drop was lower than the measured one. However, as the pressure drop change is related to mass change, the same effects, as described in the previous section, which are relevant to mass change, could explain this discrepancy.

### 3.5. Measured and Calculated Pressure Drop Changes for Mixture Aerosols

**Figure 11:**
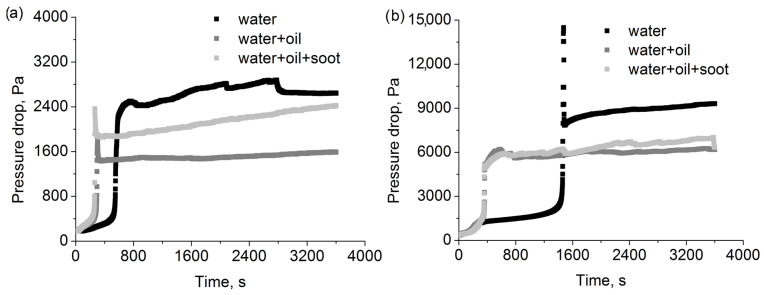
Pressure drop measured during filtration of various aerosols for (**a**) 2F6 and (**b**) F1 filters.

Figure 11 shows the change of pressure drop for 2F6 and 2F1 filters as a result of the deposition of solid and liquid aerosol particles on the fibres and in the depth of the filter. The pressure drop for filter 2F6 is clearly lower than that for filter F1 (note the different scales on the two graphs). This is due to fewer deposits being retained on this filter due to the lower efficiency of this filter. At the same time, filter 2F6 has a higher packing density, which results in a lower total volume available for deposits. The efficiency of the filter is responsible for the rate at which this volume is being exhausted, and the packing density is responsible for the total capacity of the filter towards the deposits. 

In both cases, the addition of an aerosol of oil droplets causes a significant acceleration in the filling of the layer with deposits. This results in an earlier occurrence of a rapid increase in pressure drop. According to Table 1, the mass flow of the oil-containing aerosol is two orders of magnitude lower than that of water, so the explanation for this phenomenon cannot be an increase in the mass of deposits retained on the layer in a given time unit. The reason should be considered as differences in fibre wettability by water droplets and oil droplets. The filters are made of polypropylene, which is a hydrophobic polymer. Even a small mass addition of oil to the aerosol stream improves the wettability of the fibre and makes it easier for water droplets to penetrate the filter, as well as for the deposited liquid to move through the depth of the filter. This leads to an equal distribution of the liquid inside the filter and a lower pressure drop compared to an aerosol containing only water droplets.

Despite the low mass of potential solid deposits, the addition of solid particles in the aerosol directed at the filter has a clear effect on the pressure drop, especially for the 2F6 filter. The pressure drop for an aerosol containing additional soot solid particles is significantly higher in the case of the 2F6 filter, and slightly higher (especially towards the end of the measurement) for the F1 filter. Additionally, the increase has a higher rate compared to a system containing only a mixture of liquid particles. The presence of solid particles in the aerosol and their deposition on the filter fibres lead to an increase in the efficiency of the effective capture of other aerosol particles. This effect is much less pronounced in the case of a filter which was characterised by high initial and overall filtration efficiency. 

A high peak in pressure drop followed by a decrease and stabilisation of the pressure drop value is, in fact, present in all cases for the F1 filter (for aerosol containing only water) and for the 2F6 filter (for an aerosol containing water and oil and an aerosol containing water, oil, and graphite). Its absence from the graph for the other cases is due to insufficient time resolution of the measuring system (peak duration is a few seconds at most, depending on the case). The occurrence of this peak is a result of the top layer of the filter being filled with particles, which leads to a decrease in the surface available for the aerosol flow. In an extreme case, almost the entire cross-sectional area is filled, which would lead to an increase in the pressure drop to an infinite value. However, the rapidly increasing difference, in pressure drop, between the upstream and the downstream of the filter leads to the penetration and movement of the deposits collected on the surface layer of the filter deep inside the filter. As a result, the pressure drop stabilises at a new level, resulting from the distribution of deposits throughout the filter volume. The following increase in pressure drop for the aerosol, which contains solid particles, results from their capture on the surface part of the layer, which leads to an increase in filter capacity towards the liquid particles. In the case of mixture liquid aerosol, the presence of oil particles allows for the easier movement of the captured liquid through the filter, and the resulting pressure drop change is lower than that for the pure water aerosol.

**Figure 12:**
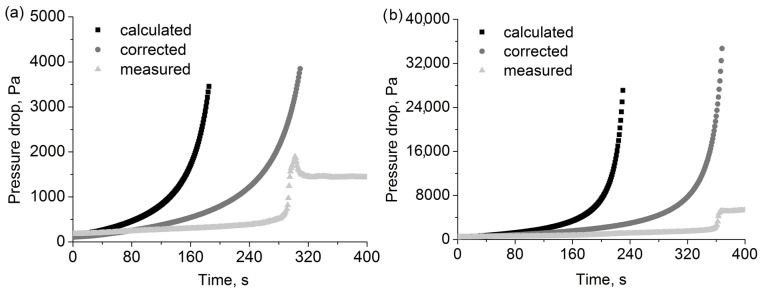
Pressure drop measured and calculated during filtration of water + oil aerosol for (**a**) 2F6 and (**b**) F1 filters.

Additionally, upon utilising the relations for the pressure drop determined for the oil aerosol, we carried out calculations for two selected filters by including in the model the presence of water droplets. The obtained results show an interesting tendency despite deviating considerably from the measured values. For both filters, in order to correct the calculated time of the occurrence of the pressure peak and match it with the corresponding measured time, it must be taken into account that only 64% of the change in packing density is effective (Figure 12). Thus, part (36%) of the predicted deposits permanently retained in the filter actually leaves the filter, either by particle resuspension or by a different kind of liquid movement inside the filter. The same value for the two tested filters, which differ significantly in their structure and characteristics, suggests that the explanation should be attributed to the nature of the tested aerosol rather than the filters. The aerosols utilised in the experiment contains particles of different phobic properties. The difference in the pressure drop values may occur due to the aforementioned mass deposit distribution inside the layer of the filter and the local distribution of flow velocity inside a partially filled filter, including the presence of the dead zones in the flow field. All of these effects were not included in the current pressure drop model.

## 4. Discussion

Taking into consideration the complete range of oil droplets, an updated filtration efficiency model with improved coefficient fitting and sizes predicts the mass change for three of the four filters with increased accuracy. Further improvements would require a consideration of the mass distribution of deposits within the filter structure, their effect on the local filter properties, and their non-uniform change over time.

The results show a major influence of aerosol composition on the pressure drop across the filter when the filter is loaded with various aerosol particles. The addition of the aerosol containing oil droplets to the aerosol containing water droplets results in an accelerated occurrence of the peak in the pressure drop and a significant decrease in the value to which the pressure drop falls after the peak. This effect can be explained by the differences in the wettability of both liquids in relation to the polypropylene from which the tested nonwoven filters have been made. In addition, in the case of a lower efficiency filter (2F6), the addition of soot particles results in a smaller decrease in peak pressure drop and a more rapid increase in pressure drop due to the deposition of particles on the filter fibres. The same increase over time can also be observed for the F1 filter. This can be explained by the increased efficiency resulting from the function of the solid particles as additional anchor points for droplets and as collection points for deposited liquid particles. The presence of these points will have a more pronounced effect in the case of the filter of lower efficiency. 

The presented calculation results comprise the first stage of building a comprehensive model of the dynamics of pressure drop on the filters during the filtration of various types of aerosols. As shown in this work, this description works best in the case of a small load of aerosol particles and/or droplets on the fibres, i.e., during the initial stage of filtration. For further stages, the difference between the results of calculations and experiments becomes much more prominent. The solution to this problem would be a further modification of the model, e.g., by dividing the considered filter into separate thin layers. This approach requires a much more detailed understanding of the distribution of deposits within the filter and the changes over time of this mass distribution. An entirely valid model should include two stages, pre-surge and post-surge of pressure drop. The first stage is an increase in resistance as a result of filling the filter space with deposits and the related mass distribution in the internal structure of the filter, which changes dynamically during filtration. The second stage is the dynamic equilibrium and is dominated by the process of the drainage and dripping of liquid deposits with minor changes, resulting from the slow filling of areas that previously remained empty, or in the case of solid particles, additional slow increase in the potential capacity of the filter due to the presence of new attachment points. In order to construct such a model, more detailed experimental observations of the mass distribution inside the filter and changes in this distribution over time should be carried out. We have no doubt that such research will be undertaken in the future. However, these studies go beyond the scope of this article, the aim of which was to present and qualitatively explain changes in flow resistance during filtration and the influence of the type of aerosol (binary solid–liquid, triple solid–liquid–liquid) on these changes.

## 5. Conclusions

An improved model based on the classical filtration theory performed better in predicting filter mass change in comparison with the previously utilised model. Improving the prediction of change in pressure drop over time would require further modifications to the model, considering the distribution of the mass of the deposits inside the filter structure.

## Figures and Tables

**Table 1:** Properties of aerosols utilised in experiments.

Aerosol	Average Particle Size, nm	Total Concentration, Particles/cm^3^	Mass Flow, g/s
soot	107.6 ± 67.8	8.46 × 10^6^	1.4 × 10^−6^
oil	248.2 ± 176.4	7.82 × 10^6^	3.3 × 10^−4^
soot + oil	201.9 ± 146.6	12.56 × 10^6^	3.3 × 10^−4^
water	3427.1 ± 1247.6	1.39 × 10^6^	2.9 × 10^−2^

**Table 2:** Filter properties.

Layer	Average Fibre Diameter, µm	Layer Thickness, mm	Average Sample Mass, g	Average Layer Mass, g/m^2^	Packing Density, -	Initial Pressure Drop, Pa
2F6	6.57 ± 3.92	2.050 ± 0.089	1.722 ± 0.061	219.3 ± 7.8	0.115 ± 0.005	160 ± 17
F6		0.878 ± 0.089	0.842 ± 0.022	107.2 ± 2.8	0.127 ± 0.012	70 ± 11
2F1	1.31 ± 0.54	3.733 ± 0.137	0.787 ± 0.042	100.2 ± 5.3	0.031 ± 0.001	550 ± 35
F1		2.128 ± 0.073	0.552 ± 0.035	70.3 ± 4.5	0.035 ± 0.001	400 ± 29

**Table 3:** The efficiency equations coefficients values.

Filter	A	B	C	D	E
2F6	0.882	1.351	0.0032	0.087	0.541
F6					
2F1	0.782	0.605	0.1479	0.125	0.294
F1					

**Table 4:** Sample mass changes during oil aerosol filtration.

Filter	Filter Mass Change, g		
	Measured	Previous Model *	Current Model
2F6	0.5097	0.3925	0.5015
F6	0.2722	0.3571	0.3768
2F1	1.1866	0.4539	0.7246
F1	1.1545	0.4536	0.7244

* results were adapted with permission from [36].

**Table 5:** The pressure drop equation coefficients values.

Filter	F	G	H
2F6	2.643	0.754	1.981
F6	1.651	0.953	2.043
2F1	1.647	0.550	1.888
F1	2.670	0.504	1.853

## Data Availability

The data presented in this study are available on request from the corresponding author. The data are not publicly available due to data being obtained based on a self-developed calculation programme which is not a part of the present manuscript.
